# Multifunctional UV and Gas Sensors Based on Vertically Nanostructured Zinc Oxide: Volume Versus Surface Effect

**DOI:** 10.3390/s19092061

**Published:** 2019-05-02

**Authors:** Leonidas E. Ocola, Yale Wang, Ralu Divan, Junhong Chen

**Affiliations:** 1 Argonne National Laboratory, 9700 S-Cass Avenue, Argonne, IL 60439, USA; ocola1962@gmail.com; 2 Department of Mechanical Engineering, University of Wisconsin-Milwaukee, 3200 N Cramer Street, Milwaukee, WI 53211, USA; yalewang@uwm.edu

**Keywords:** gas sensor, polymer infiltration, UV sensor, atomic layer deposition

## Abstract

This article reports that it is possible to make multifunctional sensing devices with ZnO infiltrated polymers while the sensing interactions could occur throughout the polymer. As such, we find that infiltrated devices with SU-8 polymer can result in highly sensitive UV sensors. Mesh dielectric core devices were found to make sensitive gas sensors with a better than 5 ppm sensitivity for formaldehyde and NO_2_. A new type of p-n junction device is further demonstrated that is sensitive to UV illumination, thus making it an enhanced UV sensor. Sensing devices relying on volume interactions, such as light absorption, can significantly benefit from the infiltrated polymer. In contrast, devices that rely on surface interactions, such as gas sensors, do not gain performance in any significant way with or without the infiltrated polymer.

## 1. Introduction

Metal oxides have been widely investigated and applied in chemoresistive gas sensors [1], photocatalysis [2,3] (Liu et al., 2013; Liu et al., 2014), optoelectronic devices [4,5], and water treatment [6,7]. Zinc oxide (ZnO) applied in these applications takes the form of nanoparticles, nanorods, or thin films. These crystalline or polycrystalline metal oxides are grown via chemical synthesis or physical deposition and have been extensively characterized [8,9]. In this paper, a different growth method of ZnO inside a polymer film is studied. The large bandgap and semiconductor properties of ZnO allow for a visible-blind ultraviolet light sensor.

ZnO nanoparticles or quantum dots have been incorporated inside polymers for many years. Their optical properties have been combined to develop new applications, like nonlinear optics [10], photovoltaic applications [11,12], and UV-Vis absorbers [13]. ZnO could be combined with a transparent matrix to form suitable UV-shielding materials, filters, and luminescent films. With the increased portfolio of materials that can be deposited using atomic layer deposition (ALD), there has been an increased interest in infiltrated metal oxides [14], such as zinc oxide, in terms of the fundamental understanding of growth properties in resistive polymers, such as poly (methyl methacrylate) (PMMA) [15] and the negative resist SU-8 [16]. The concepts of the infiltration method are similar to the ALD process. ALD is a vapor phase deposition technique with precise thickness control and high uniformity [17]. The infiltration method is a variant of ALD specifically to coat inside walls of porous materials, but the process exposure time, pressure, and purpose are significantly different. The purpose is to allow the precursor gases to infiltrate the polymer matrix (e.g., SU-8) and the reaction to occur inside the polymer matrix. 

Introduction of infiltrated ZnO in SU-8 for novel device fabrication has also been addressed in prior work [18,19]. SU-8 is a negative resist that can be exposed with UV light at 385 nm and also with high energy electron beams. In the work by Nam et al., the SU-8 polymer was removed via oxygen plasma etch after infiltration and left behind pure ZnO nanowires [18,19]. This report discusses the effect of leaving the infiltrated polymer as part of the fabrication of transparent UV and gas sensor devices. The advantages of infiltration over simple ALD coating as part of the fabrication process has been investigated.

## 2. Experimental

### 2.1. Energy-Dispersive X-Ray Spectroscopy (EDS) Characterization

**Figure 1:**
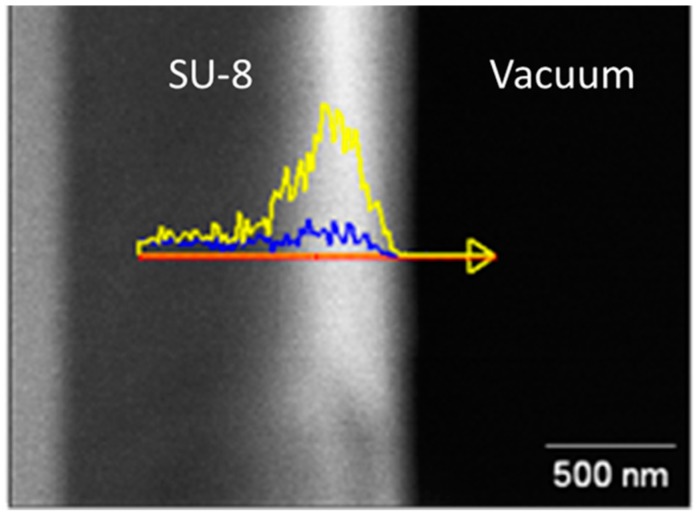
Energy-dispersive X-ray spectroscopy (EDS) data of the L-shell for Zn (yellow) from infiltrated ZnO and the K-shell for Al (blue) from the Al_2_O_3_ seed layer in SU-8 (cross sectional view).

The penetration depth of the infiltration process in SU-8 was determined by infiltrating an SU-8 film that was first flood-exposed to create a uniform 1 μm-thick thin film that would allow energy-dispersive X-ray spectroscopy (EDS) cross section characterization. The sample was infiltrated with 12 cycles of water and diethylzinc [H_2_O:DEZ], then cleaved and tilted to expose the lateral side to an electron beam in a field emission scanning electron microscope JEOL 7500 FESEM. Each half cycle of H_2_O and DEZ lasted 4 min and processed at 95 °C. Figure 1 shows the EDS data of the L-shell for Zn and the K-shell for the Al_2_O_3_ seed layer used to enhance ZnO formation inside the polymer [11]. The data shows that the penetration depth can reach about 500 nm into the resist. This means that if the fabricated SU-8 structures are on the order of 1 μm in width, then it is reasonable to expect that the entire structure will be saturated with infiltrated ZnO. Another detail can be assessed from Figure 1, in which there is no evidence of ZnO nanoparticle formation, where polymer nanoparticle interfaces can be seen. It is thus reasonable to expect a uniform distribution of ZnO in the infiltrated region. A comprehensive investigation on the growth of zinc oxide in a polymer matrix using photoluminescence (PL), Raman, and X-ray photoemission spectroscopy (XPS) has been reported in our previous paper [15].

### 2.2. Sensor Fabrication

The device under test is a novel design with an enhanced surface area for both light absorption and gas sensing [20]. It is based on locating the sensing material over a set of interdigitated electrodes that cover a 20 μm × 200 μm area with electrode fingers 300 nm wide and placed at a 1 μm pitch. The use of interdigitated electrodes covered with the sensing metal oxide material has been shown to render high sensitivity and good performance [21].

**Figure 2:**
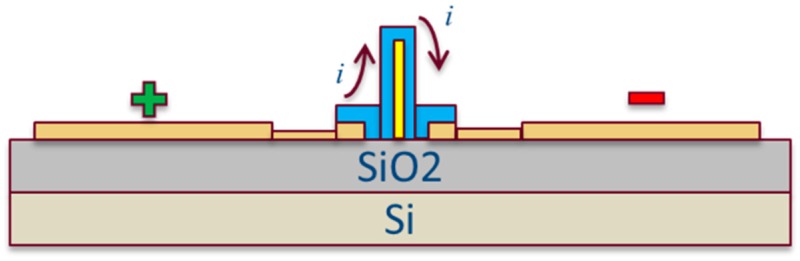
Schematic of the device structure of the UV and gas sensor. A vertical dielectric core (HSQ) is surrounded by infiltrated polymer (SU-8) that connects two electrodes (+ and −), forcing the current (***i***) to go up and over the dielectric core.

A schematic of the device to be discussed is illustrated in Figure 2. We locate a dielectric core surrounded by infiltrated polymer that connects two electrodes. The dielectric core forces the electric current between electrodes out of the plane and into the sensing media. It is made by patterning 1 μm-thick hydrogen silsesquioxane (HSQ) resist with 100 KV electrons at a dose of 7000 μC∙cm^−2^, and developed in a 1:3 diluted solution of Microposit 351 developer in water for 5 min. After the fabrication of the dielectric core, 1.5 μm of SU-8 was spin coated, baked at 100 °C for 3 min, exposed at a dose of 28 μC∙cm^−2^ at 100 KV, and developed in an SU-8 developer for 2.25 min. The SEM layout images of the device are indicated in Figure S1 in Supporting Information.

## 3. Results and Discussions

**Figure 3:**
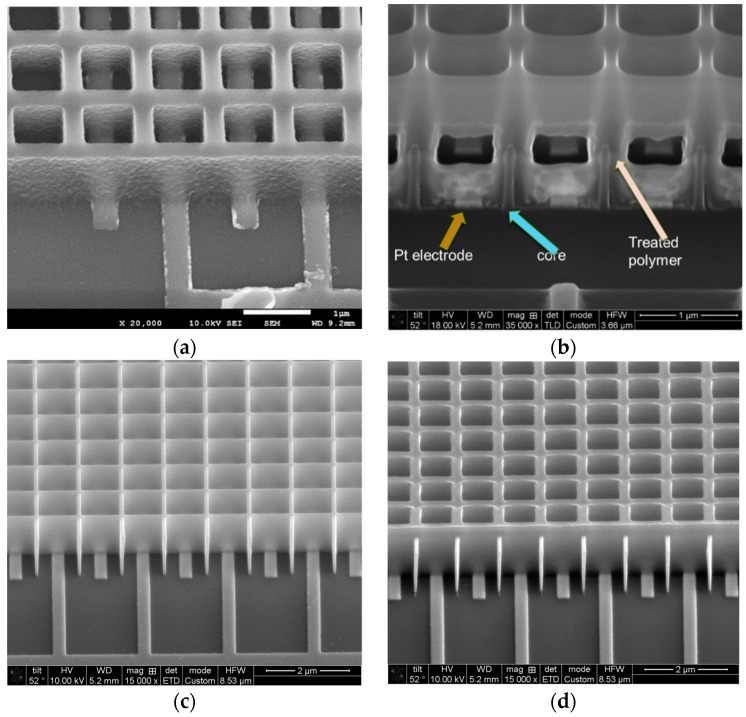
SEM micrographs showing key aspects of a fabricated device after infiltration. (**a**) Image shows a 1 μm tall–300 nm wide mesh structure over a set of interdigitated electrodes. (**b**) Image of the same device after focused ion beam (FIB) cross sectioning exposing the dielectric core, Pt electrodes, and infiltrated SU-8 polymer. (**c**,**d**) Devices fabricated with extra dielectric core fins to ensure electrical current goes over the core into the sensing media (**c**) device with only a core and no polymer, and (**d**) device with core and polymer.

A first device was fabricated with 12 cycles of infiltrated ZnO as shown in Figure 3a,b. The sample was cured at 400 °C to improve the ZnO crystallization and thus improve the current.

A second generation of devices with an improved design to ensure that all current between electrodes does indeed enter the sensing media were fabricated and are shown in Figure 3c,d. In addition, these devices were used to determine if adding the polymer for infiltration improved the performance of the device or not. Therefore, both devices received the exact same infiltration treatment, thermal annealing (in this case, at 300 °C in an oxygen-rich atmosphere). The lower temperature was used to minimize any chance of polymer degradation during annealing. In this case, it could be drawn at low voltages to maximize the current, the samples were subjected to 36 cycles of infiltrated ZnO, plus a coating of a ZnO and a TiO_2_ bilayer of 36 cycles each. TiO_2_ was used for a dual purpose. First, TiO_2_ helps stabilize ZnO [2], and second, the heterostructure formed between ZnO and TiO_2_ facilitates charge separation [22]. In order to ensure there was no difference in the processing of these devices, they were both fabricated on the same silicon substrate and infiltrated in the same chamber while on the same wafer substrate. Therefore, any performance difference was only due to the presence or not of the polymer.

Given that infiltration is an ALD-based technique, it is expected that the ZnO will coat the entire wafer. Therefore, before any sensing experiments, the ZnO was etched back from all areas with the exception of the sensing region in the mesh region. This prevents shorts between the electrodes and forces the electrical current through the out-of-plane mesh structures, thus enhancing the interaction of the electrical current in the metal oxide with the surrounding media.

### 3.1. Response to UV Light

**Figure 4:**
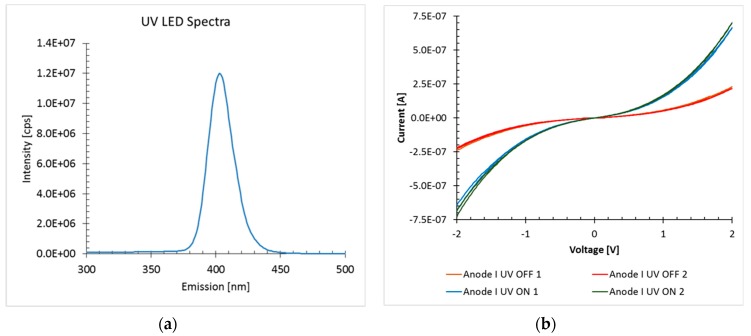
First UV sensing results with infiltrated ZnO. (**a**) Spectral range of the UV LED used. (**b**) Current vs. voltage (I-V) curve with the UV Light-Emitting Diode (LED) turned off and on (dark line going from −650 nA to +650 nA at ± 2V).

ZnO has been widely investigated as visible and ultraviolet (UV) light photodetection materials for decades. The first structure of the infiltration ZnO in a polymer matrix with the vertical dielectric core increased the sensing surface area compared with the thick ZnO film. The initial results using this first device as a UV sensor are shown in Figure 4. The current as a function of the driving voltage with a UV Light-Emitting diode (LED) turned off and on was measured. The spectral range of the UV LED is shown in Figure 4a, while the I-V curve is shown in Figure 4b. The device was able to achieve a current of 650 nA at 2 V under 408 nm UV light illumination.

**Figure 5:**
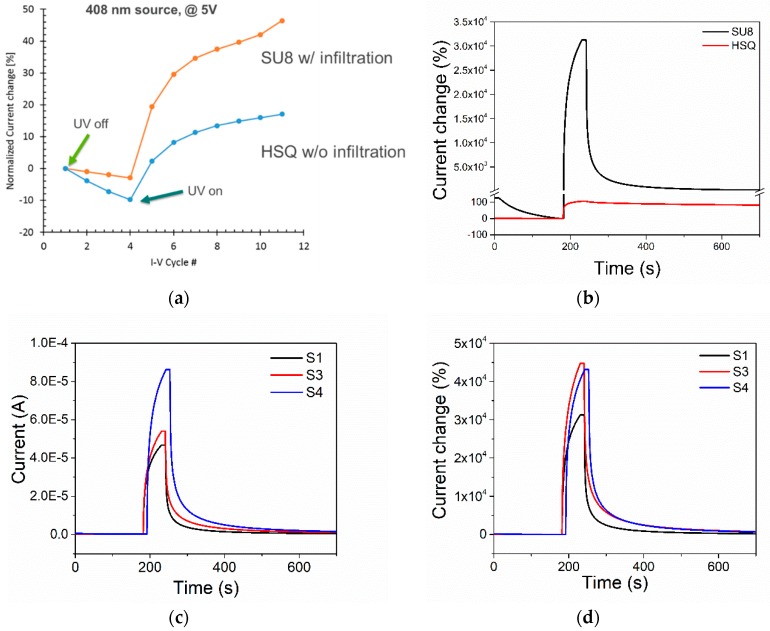
(**a**) Change of current at 5 V when turning on UV LED at 408 nm in 30 s intervals for devices with the hydrogen silsesquioxane (HSQ) dielectric core with/without w/o infiltration, and with photoresist polymer (SU-8) with infiltration. (**b**) Change of current vs. time when turning on UV lights for devices with a HSQ dielectric core, and with SU-8 polymer. (**c**,**d**) Change of current vs. time for infiltrated devices with SU-8 polymer taken at 2 V. S1 recipe: H_2_O, BA-MeOH, H_2_O; S3 recipe: (DEZ, H_2_O) × 2 cycles, BA-MeOH, H_2_O; S4 recipe: (DEZ, H_2_O) × 2 cycles, (BA-MeOH, H_2_O) × 2 cycles. (**c**) Current vs. time showing actual current in Amps. (**d**) Percentage change of current vs. time normalized to the average current before turning on the UV light source.

The comparison of the UV sensing performance between devices is shown in Figure 5a. The data is the current measured at 5 V after cycling the electrode voltage from −5 V to +5 V with the UV LED source at 408 nm off and on. The time for a voltage sweep took about 30 s. Figure 5b demonstrates the current change of the SU8 and the HSQ devices as a function of time. As can be seen on the plot, the device with the polymer has a stronger change in the current than the device made with only the dielectric core. This can be explained as the UV light is absorbed by the full device, including the coating polymer and infiltrated ZnO. A second test using a 365 nm UV source generated by a UV-AC lamp purchased from VWR, which is more energetic, shows that the infiltrated devices with polymer have a sub 10 s response 200 to 300 times (20,000% to 30,000%) change in current, Figure 5c,d. The UV photoresponse current was monitored by a Keithley 2602 source meter at 2 V bias. The current at 2 V was of the order of tens of nA with the 365 nm UV light off. Then, it jumped to the order of tens of μA with the UV light on. Data are normalized to the average current measured before the UV light was turned on. The three data sets in Figure 5 correspond to the different fabrication recipes.

Figure 5d indicates the UV sensitivity ($\frac{I_{U}-I_{a}}{I_{a}}$) of the S3 sensor reached 4.5 × 10^4^, which is comparable with the highest ratio (1 × 10^4^ to 1 × 10^5^) reported in the literature [23,24]. $I_{U}$ represents the current with the UV light turned on and $I_{a}$ represents the current with the UV light turned off. We also investigated the stability performance of the UV sensor in Figure S2.

**Figure 6:**
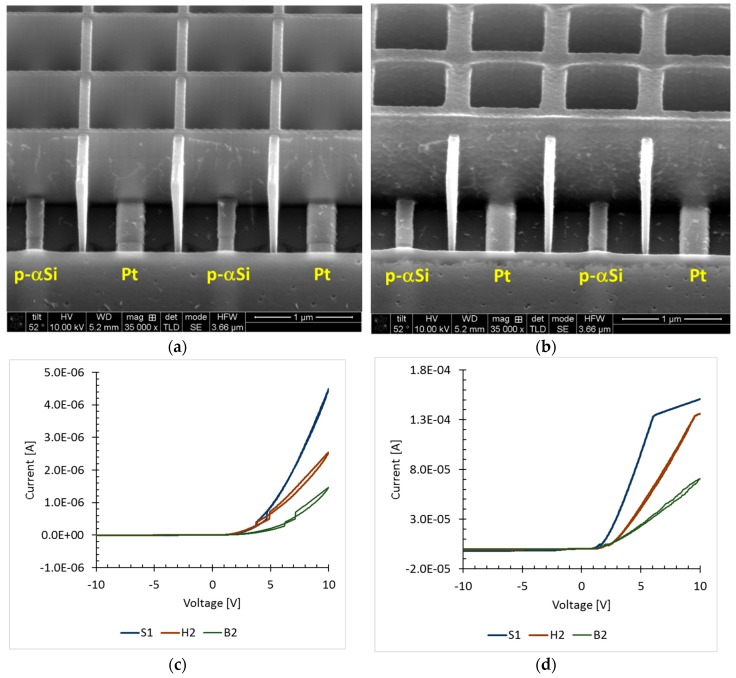
SEM images of the p-n junction devices with coated or infiltrated ZnO. (**a**) Device with dielectric core coated with ZnO. (**b**) Device with ZnO infiltrated SU-8 polymer. Current vs. voltage plots of p-n junctions with coated or infiltrated ZnO. Device S1 corresponds to a device with ZnO infiltrated SU-8 polymer. Device H2 corresponds to a ZnO coated dielectric core device. Device B2 corresponds to a non-patterned device where ZnO is coating the electrodes directly with no dielectric core mesh. Note the difference in scales between UV off data (**c**) and UV on data (**d**) is almost two orders of magnitude.

To create a p-n junction with the coated and infiltrated ZnO, one of the interdigitated platinum electrodes was replaced by a boron-doped patterned amorphous silicon electrode, and images of the devices used are shown in Figure 6a,b. Preliminary results clearly show rectification by the devices with and without UV light exposure, Figure 6c,d. It is apparent that the p-n junctions with ZnO infiltrated SU-8 polymer are more conductive than its counterparts. It is believed that this is due to the conduction below the polymer surface, which contributes to the total conductivity. The kink in the high current region is believed to be an artifact from the acquisition hardware.

### 3.2. Response to Formaldehyde and NO_2_

The gas sensing properties with the same devices were investigated. The gas sensing test system consists of a home-made chamber, a Keithley 2602 source meter for current monitoring and source-drain voltage application, and two mass flow controllers connected to air and testing gas cylinders. The first generation of devices was tested for formaldehyde and NO_2_. As a background, the recommended formaldehyde gas exposure limits are: STEL: 0.3 (ppm) from ACGIH (TLV) (United States), STEL: 2 (ppm) from OSHA (PEL) (United States), STEL: 2 (ppm) (United Kingdom (UK)) [25]. The exposure limits for NO_2_ are 5 ppm OSHA ceiling, 1 ppm OSHA STEL, 3 ppm ACGIH TWA, 5 ppm ACGIH STEL, and 1 ppm NIOSH recommended STEL [26]. STEL is short-term exposure limit, TLV is threshold limit value. 

**Figure 7:**
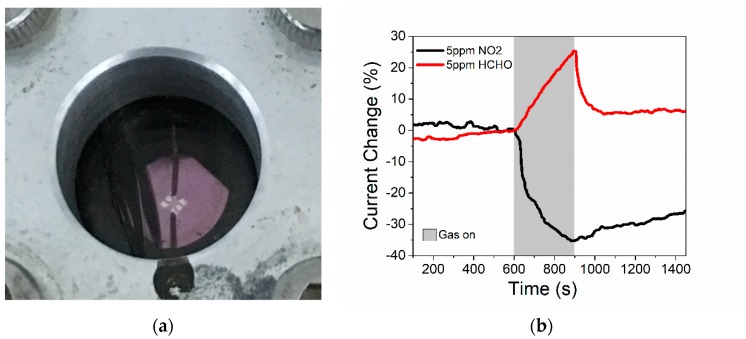
Gas sensing application of infiltrated devices. (**a**) First generation device in a gas sensing chamber. (**b**) Data showing the change of current (%) as a function of time as the device is exposed to 5 ppm of formaldehyde and 5 ppm of NO_2_.

These chemiresistive gas sensors responded to analyte gases with a current change due to the carrier density modification effect from the gas molecule interaction with the sensing material surface. The sensitivity is defined as $S=\frac{\left(I_{g}-I_{a}\right)}{I_{a}}$, where I_g_ represents the current in gas and I_a_ represents the current in air. The first test and Figure S3 indicate the ability of the devices to detect sub 5 ppm NO_2_ gas, Figure 7. The literature has reported on the polymer/ZnO sensor for NO_2_ sensing. Chougule and his group synthesized the polypyrrole-ZnO nanocomposite gas sensor [27]. The sensitivity of the sensor against 100 ppm NO_2_ was around 37% at room temperature. Our ZnO-SU8 sensors responded with 36% variation in the sensing signal against much lower concentrations of NO_2_ (5 ppm). Wang reported the poly(3-hexylthiophene) (P3HT)/ZnO hybrid NO_2_ sensor [28]. The sensitivity reached 55% against 4 ppm NO_2_ gas, which is slightly higher than the sensitivity reported here because pure P3HT showed sensitivity against NO_2_. However, pure HSQ and SU8 polymer did not respond to NO_2_ gas. 

The current responses also suggest that the ZnO is n-type as formaldehyde is a reducing gas while NO_2_ is an oxidizing gas. The mechanism of sensing gases by n-type ZnO can be explained by the oxygen ions’ surface sorption model [29]. At room temperature, oxygen molecules from air are adsorbed on the surface of the zinc oxide. Carriers (electrons for ZnO) are transferred from the conduction band of the n-type oxide semiconductor to the adsorbed oxygen atoms, and at room temperature, the dominant specie is O_2_^−^. During the exposure to NO_2_ gas, the gas molecules are adsorbed on the ZnO surface. NO_2_, as an oxidizing gas, acts as an acceptor of electrons (2NO_2_ + O_2_^−^ + 2e^−^ → 2NO_3_^−^) thus reducing the current [30]. When the metal oxide surface is exposed to formaldehyde gas, O_2_^−^ reacts with the gas molecules and the electrons are released back to the conduction band (HCHO + 2O_2_^−^ → CO_2_ + H_2_O (g) + 4e^−^), thus increasing the current [31]. When the surface-to-volume ratio is high, more O_2_^−^ are adsorbed on the material surface, and the reaction between gas molecules and O_2_^−^ species increases the sensitivity [32].

**Figure 8:**
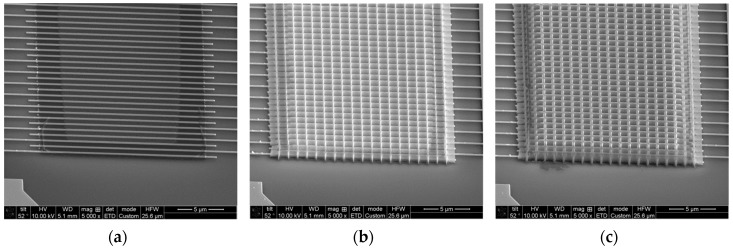
Set of devices to test the effects of the surface area, and polymer infiltration on the performance for gas sensing. (**a**) Thin film of ZnO over the interdigitated electrodes and no dielectric core mesh. (**b**) Dielectric core mesh coated with ZnO. (**c**) SU-8 polymer with interior dielectric core mesh infiltrated with ZnO.

A dielectric core only device performance was compared with the performance of a device with infiltrated SU-8 polymer. In addition, a device with no mesh pattern was added so it is possible to determine if the increased mesh surface area has a significant effect on the device gas sensing performance, Figure 8. 

**Figure 9:**
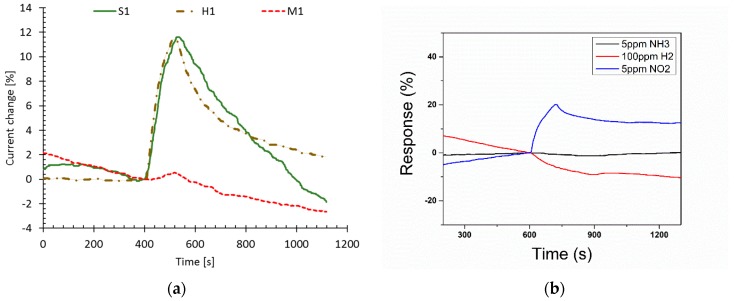
(**a**) Gas response plots to 5 ppm of NO_2_ for the three types of devices as shown in Figure 8. Data labeled M1 corresponds to a device with no mesh. Data labeled H1 corresponds to a device with a dielectric core mesh. Data labeled S1 corresponds to a device with a dielectric core mesh and infiltrated SU-8 polymer. Data were normalized to the device response right before gas was turned on at 400 s. (**b**) Gas response plots to 5 ppm NH_3_, 5 ppm NO_2_, and 100 ppm H_2_ of the S1 sample.

The gas response results are shown in Figure 9a. It is clear from the data that the extra SU-8 polymer does not improve the response to the gas. The reason is that the interaction of the device to the gas is a surface effect. The polymer device has the same surface area as the dielectric core device. Figure 9 indicates that the device baseline during the gas sensing is not flat. We believe it is a thermal effect during measurement. The selectivity performance was investigated as shown in Figure 9b. The polymer device indicated a larger current change (21%) in NO_2_ than in NH_3_ (~2%) or H_2_ (~10%) at same or even lower concentrations. We also found the device could detect NO_2_ gas lower than a 5 ppm concentration (Figure S3).

## 4. Summary and Conclusions

This report has demonstrated that it is possible to make interesting sensing devices with infiltrated polymers when the interactions penetrate into the volume of the polymer. As such, we find that infiltrated devices with SU-8 polymer can make sensitive UV sensors. We also found that mesh dielectric core devices can make sensitive gas sensors with better than a 5 ppm lower detection limit. The infiltrated devices could respond to 5 ppm NO_2_ with a ~12% current change. There is still room for further understanding of the interactions of the ZnO and the gases and improvement in the baseline stability of the device. A new type of p-n junction device that is highly sensitive to UV illumination was demonstrated, thus making it a more sophisticated UV sensor. We showed that devices with the infiltrated polymer can serve as transparent multifunctional sensors that are responsive to both UV and gases, with UV sensing throughout the volume of the infiltrated polymer while gas sensing occurs at the polymer surface. 

## Supplementary Materials

The following are available online at https://www.mdpi.com/1424-8220/19/9/2061/s1, Figure S1: SEM images of the sensor layout, Figure S2: Multi-cycled UV response curve of the ‘S’ sample, Figure S3: Gas response plots to 1 ppm NO2 of S3 and S4 samples.
