# Supplementary material for: Multifunctional UV and Gas Sensors Based on Vertically Nanostructured Zinc Oxide: Volume Versus Surface Effect
Source: Sensors (Basel). 2019 May 2;19(9):2061. doi: 10.3390/s19092061 (PMC6539821; doi:10.3390/s19092061)
Supplement: Supplementary file 1 [file sensors-19-02061-s001.pdf]

Supporting information

Multifunctional UV and gas sensors based on vertically nanostructured zinc oxide: volume versus surface effect

Leonidas E. Ocola, Yale Wang, Ralu Divan and Junhong Chen

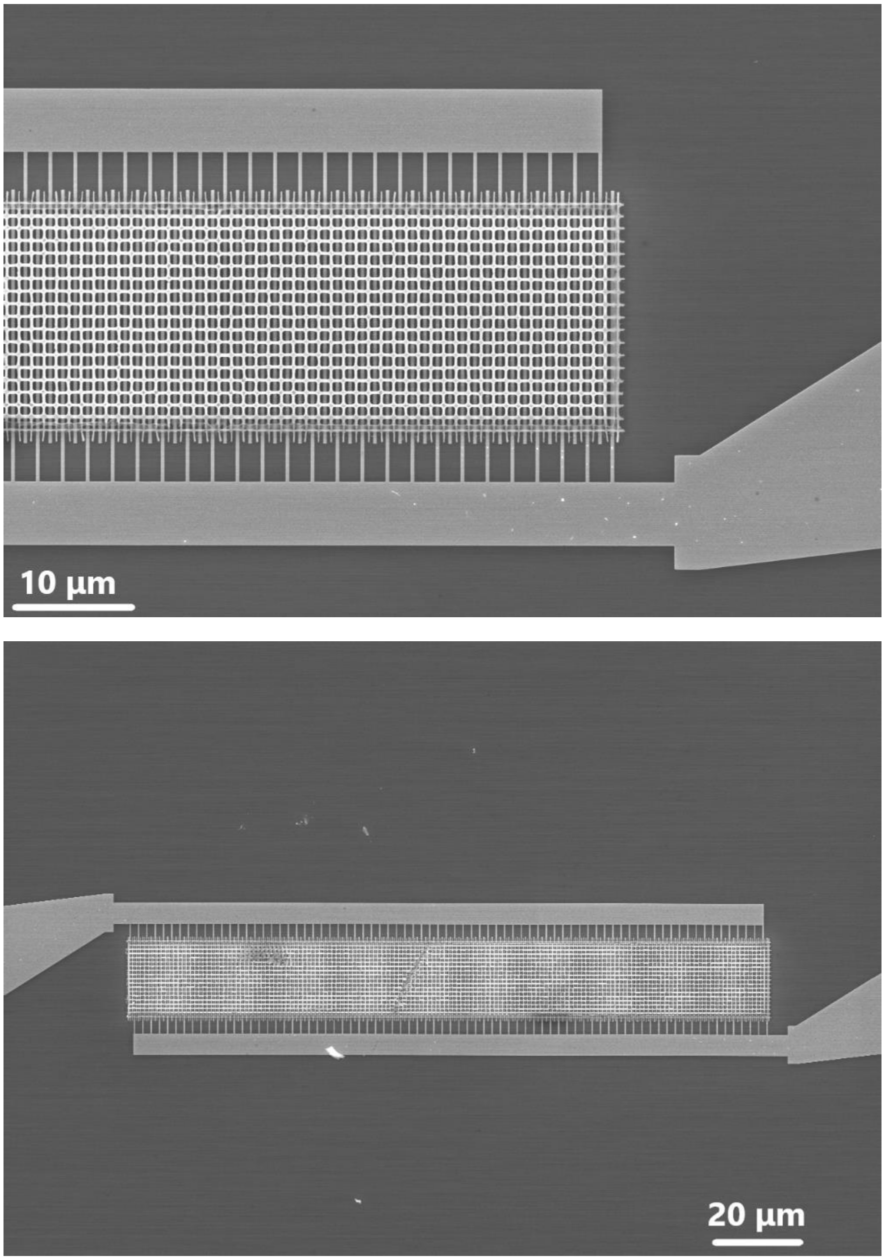

Figure S1. SEM images of the sensor layout.

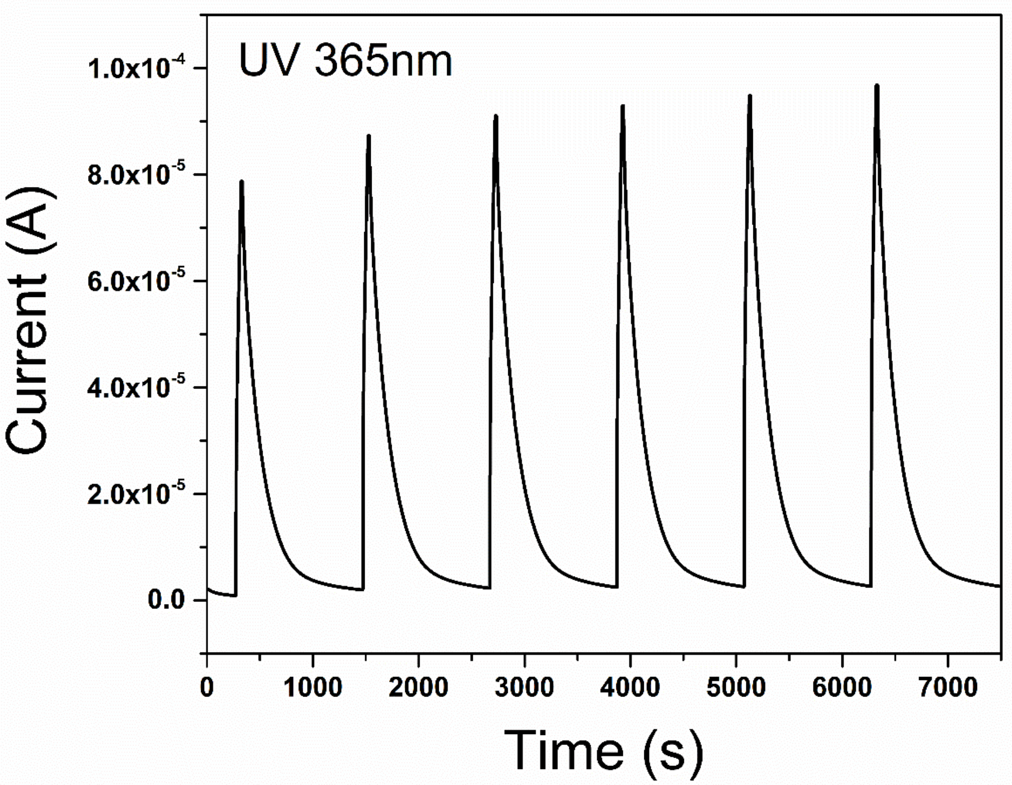

Figure S2. Multi-cycled UV response curve of the 'S' sample.

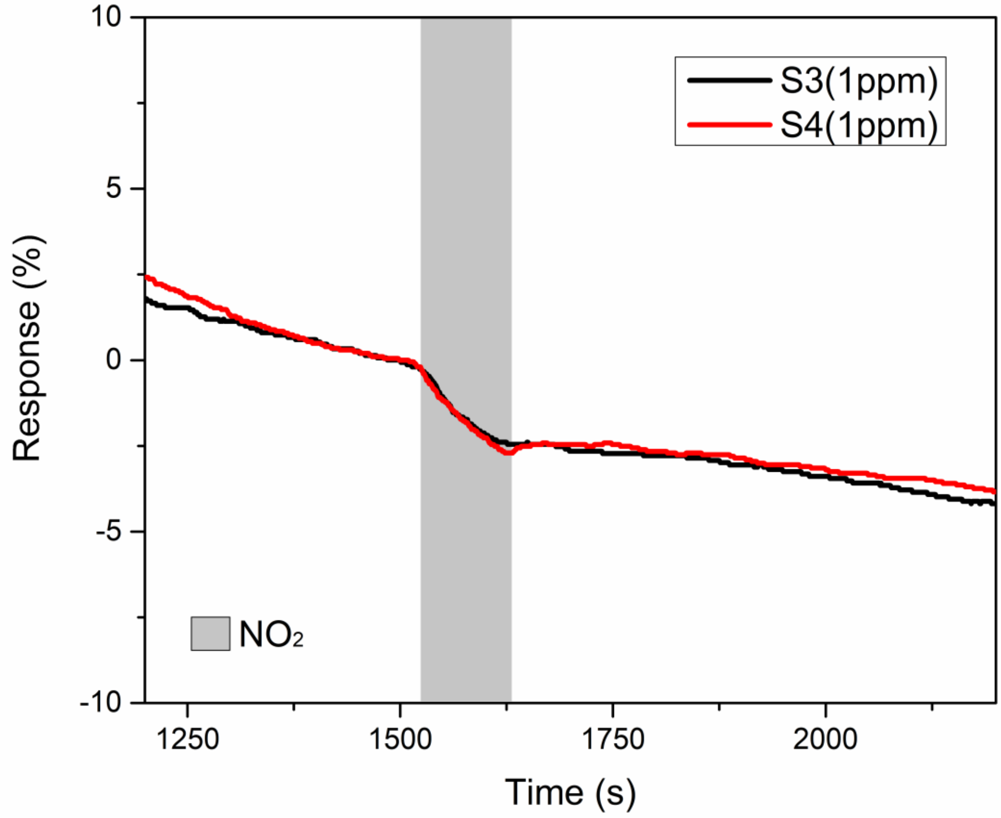

Figure S3. Gas response plots to 1 ppm NO_2 of S3 and S4 samples.
